# Clinico-Biochemical Correlation in Birth Asphyxia and Its Effects on Outcome

**DOI:** 10.7759/cureus.11407

**Published:** 2020-11-09

**Authors:** Abhilipsa Acharya, Banashree Swain, Sarbeswar Pradhan, Pradeep K Jena, Nirmal K Mohakud, Arakhita Swain, Niranjan Mohanty

**Affiliations:** 1 Pediatrics and Neonatology, Institute of Medical Sciences and Sum Hospital (IMS and Sum Hospital) Siksha 'O' Anusandhan (SOA) Deemed to be University (DU), Bhubaneshwar, IND; 2 Biochemistry, Sriram Chandra Bhanj (SCB) Medical College, Cuttack, IND; 3 Pediatrics, Kalinga Institute of Medical Sciences, Kalinga Institute of Industrial Technology (KIIT) Deemed to be University (DU), Bhubaneswar, IND; 4 Pediatrics, Sriram Chandra Bhanj (SCB) Medical College, Cuttack, IND; 5 Pediatrics, Kalinga Institute of Medical Sciences, Bhubaneswar, IND

**Keywords:** hypoxic ischemic encephalopathy, birth asphyxia, prolonged labor, hyponatremia, hyperkalemia, hypothermia, instrumental delivery

## Abstract

Background

Birth asphyxia is a major cause of early neonatal death and leads to severe consequences such as epilepsy, cerebral palsy, and developmental delay. This study aims to determine the correlation between dyselectrolytemia and the degree of hypoxic-ischemic encephalopathy (HIE) and to find out major risk factors contributing to the severity of HIE and neonatal death.

Methods

In this prospective cohort study (n=150), term babies weighing ≥ 2.5 kg at birth, with the diagnosis of birth asphyxia, admitted in a medical college in Odisha state from September 2014 to August 2016 were included. Clinical findings, biochemical parameters, treatment, and outcome of HIE babies were recorded.

Result

The majority of the asphyxiated babies were having moderate HIE (HIE II) (57.33%), whereas mild and severe stages were seen in 15.33%, and 27.34% of babies, respectively. Factors like prolonged labor (87.8%) and meconium-stained liquor (63.4%) were mostly attributed to the severe degree of birth asphyxia (p < 0.001). Apnea, lethargy, and hypothermia were the most remarkable feature of HIE III. The degree of hyponatremia, hypocalcemia, and hyperkalemia (124.4±4.4 mmol/l, 0.83±0.08 mmol/l, and 6.17± 0.89 mmol/l, respectively) were more severely affected in HIE III as compared to HIE l (137.5±3.8 mmol/l, 1.06±0.17 mmol/l, and 5.0±0.79 mmol/l, respectively). Serum urea and creatinine increased proportionately with an increase in the severity of HIE grade. The mildly asphyxiated neonates recovered completely, whereas all the cases who died (n=29,19.3%) belonged to the moderate or severe degree of birth asphyxia.

Conclusion

The asphyxiated neonates had hyponatremia, hypocalcemia, hyperkalemia, raised serum urea, and creatinine and correlated with the severity of birth asphyxia. Prolonged labor and meconium-stained liquor were the most attributable factor for the severe degree of birth asphyxia. Effective neonatal resuscitation and quick correction of electrolyte imbalances will help in the reduction of neonatal mortality and long-term neurological sequelae.

## Introduction

Perinatal asphyxia/birth asphyxia is a major cause of neonatal morbidity and mortality in developing countries [1]. Effective neonatal resuscitation not only saves the life of newborns but prevents long-term neurological sequelae. Perinatal asphyxia constitutes 28.8% of neonatal mortality and 45.1% of stillbirths in India [2]. Perinatal asphyxia causes hypoxia (lack of oxygen) or inadequate perfusion (ischemia) to various organs of the fetus or newborn. Birth asphyxia is defined as a combination of hypoxia, hypercarbia, and metabolic acidosis due to the blockage of umbilical vessels, placental insufficiency in utero, or ineffective breathing after delivery [3-4]. It is an insult to the fetus or newborn either in the antepartum or intrapartum period or both, leading to various functional and/or biochemical changes. Birth asphyxia is the most common preventable cause of central nervous system (CNS) injury in neonates and thereby protects from long-term neurological abnormality and disability [5].

Around 3.6 million neonates suffer from moderate to severe birth asphyxia in developing countries. Nearly 840,000 babies (23%) die or may develop serious neurological sequelae [6]. Prevention is more important than treatment for perinatal asphyxia. Maternal factors like prolonged labor, gestational diabetes, antepartum hemorrhage, preeclampsia, and multiple pregnancies have a significant role in the development of neonatal asphyxia [1]. The prolonged second stage of labor underlies around 60% of HIE and could be prevented by institutional setup and the availability of skilled medical personnel and facilities for operative deliveries, where required [7-8]. Neonatal factors for birth asphyxia (HIE) are post-dated, cord around the neck, oligohydramnios, meconium-stained amniotic fluid (MSAF), malpresentation, etc. [8]. The serum level of electrolytes plays a pivotal role in the outcome of these HIE babies. Any deviation from the normal levels of electrolytes (sodium, potassium, and calcium) may lead to convulsions, shock, and other types of metabolic abnormalities. Perinatal asphyxia results in anaerobic metabolism, decreased adenosine triphosphate (ATP) production, impairment of the function of ion pump, and the accumulation of intracellular sodium, chloride, water, calcium, and extracellular potassium leading to an electrolyte imbalance, which adversely affects the outcome of asphyxiated babies [9-10]. Besides CNS, the kidney is the most sensitive organ to hypoxia [10]. Birth asphyxia results in ischemia to the proximal tubule, thereby developing acute tubular necrosis and acute renal failure. This leads to an increase in serum urea and creatinine [10]. So the meticulous management of body temperature, electrolytes, and blood sugar and providing appropriate oxygen may reduce the severity of the ischemic insult.

The objective of the present study is to identify the risk factors and biochemical derangements associated with increased morbidity and mortality.

## Materials and methods

This prospective observational study was conducted at the department of pediatrics, Sriram Chandra Bhanj (SCB) Medical College, Cuttack, in the state of Odisha in the eastern part of India over a period of two years, from September 2014 to September 2016. The study cohort (n=150) consisted of hospitalized term neonates of birth weight ≥ 2.5 kg with the diagnosis of birth asphyxia as per the following criteria [11-12]: metabolic acidosis (cord or infant blood in 1st hour); APGAR (appearance, pulse, grimace, activity, and respiration) score < 7 at five minutes of birth; apnea at birth; base deficit > 16 mmol/l; and clinical evidence of encephalopathy (altered consciousness, seizure, hypotonia, or absence of suck).

Newborn babies with congenital anomalies, suspected inborn errors of metabolism, congenital infection, septic shock, intrauterine growth restriction (IUGR), received diuretics prior to the evaluation, and < 37 weeks of postmenstrual age were excluded.

Data of neonates presenting with perinatal asphyxia were evaluated using a prestructured proforma. Classification of mild (HIE I), moderate (HIE II), and severe (HIE III) stage was based on the modified Sarnat staging for neonatal encephalopathy [13]. Detailed history regarding type and place of delivery, type of resuscitation required, any complication before/during delivery, onset and duration of seizure, and findings of clinical examination, with special reference to the central nervous system, were noted. Various risk factors like prolonged labor, preeclampsia, antepartum hemorrhage (APH), multiple pregnancies, gestational diabetes (GDM), cord around the neck, meconium-stained amniotic fluid (MSAF), oligohydramnios, and malpresentation were recorded. Biochemical parameters like sodium (Na+), potassium (K+), calcium (Ca+2), serum urea, and creatinine, quantitative C-reactive protein (CRP), and complete blood count were estimated. Clinical findings during the treatment in the hospital until discharge or death were recorded. Neurological findings like abnormal movements, feeding problems, subtle/frank seizures, or neurological deficits were evaluated.

Data were analyzed using the Statistical Package for the Social Sciences (SPSS) version 20 (IBM Corp., Armonk, NY). A p-value of < 0.05 was considered statistically significant. Association of risk factors and biochemical parameters with respect to the severity of HIE were evaluated using the chi-square test. Furthermore, univariate analysis was used to find out the significance of various parameters in the outcome of the present study. Institutional ethical clearance was taken prior to the study.

## Results

Male babies (n=116, 77.3%) outnumbered female babies and the ratio was 3.4: 1. Term and post-term babies constituted 86 (57.3%) and 64 (42.7%) cases, respectively. The majority of babies (57.33%) were having moderate HIE, whereas 15.33% and 27.34% of babies were mild and severe HIE, respectively. Post-term babies were more likely to be affected by HIE I or HIE II as compared to HIE III (p < 0.01). Normal and assisted delivery (122/137, 89%) cases were mostly having HIE II/HIE III, whereas it was less in lower segment cesarian section (LSCS) delivery (5/13, 38.4%). Among the risk factors, prolonged second stage of labor (n=95, 63.3%) and meconium-stained liquor (n= 63,42%) were more common. The association of these two factors increased the severity of birth asphyxia (p< 0.001) (Table 1).

**Table 1:** Correlation of risk factors with the severity of hypoxic-ischemic encephalopathy HIE, hypoxic-ischemic encephalopathy; vs, versus; GDM, gestational diabetes; MSAF, meconium-stained amniotic fluid; APH, antepartum hemorrhage

Risk Factors	Mild cases (%) (HIE I)	Moderate cases (%) (HIE II)	Severe cases (%) (HIE III)	Total cases (%)	HIE I vs HIE II P-value	HIE I vs HIE III P-value	HIE II vs HIE III P-value
Prolonged labor	12 (52.2)	47 (54.7)	36 (87.8)	95 (63.3)	0.832	0.001	0.0002
Preeclampsia	1 (4.3)	2 (2.3)	3 (7.3)	6 (4)	0.598	0.637	0.387
GDM	1 (4.3)	3 (3.5)	2 (4.9)	6 (4)	0.845	0.637	0.706
Multiple Pregnancy	1 (4.3)	2 (2.3)	2 (4.9)	5 (3.3)	0.598	0.923	0.820
MSAF	1 (4.3)	36 (41.9)	26 (63.4)	63 (42)	0.001	0.0001	0.023
Cord around neck	1 (4.3)	10 (11.6)	9 (22)	20 (13.3)	0.522	0.133	0.127
Oligohydramnios	2 (8.7)	7 (8.1)	8 (19.5)	17 (11.3)	0.931	0.432	0.063
Malpresentation	3 (13)	23 (26.7)	5 (12.2)	31 (20.7)	0.273	0.921	0.064
APH	1 (4.3)	6 (7)	7 (17.1)	14 (9.3)	0.647	0.278	0.079
No-Risk Factors	8 (34.8)	11 (12.8)	0	19 (12.7)	0.013	0.0003	0.039

Other maternal factors like antepartum hemorrhage, gestational diabetes, and preeclampsia had less contribution to the development of HIE (17.3%). Babies delivered outside the hospital (n=42, 28%) had more chance of developing severe birth asphyxia ( p < 0.003). Hypothermia (n=55, 36.7%), lethargy (n= 128, 85.3%), respiratory distress (n=71, 47.3%), convulsion (n=100, 66.7%), and apnea (n=48, 32%) were associated with HIE, but apnea, lethargy, and hypothermia were the most remarkable features of HIE III.

Hyponatremia (62, 41.3%) was predominately found in HIE II/III as compared to HIE l (p<0.0001). The incidence of hypocalcemia (n=118, 78.7%) observed in HIE II/III was significant compared to HIE I (p<0.0001) (Table 2).

**Table 2:** Electrolyte levels and their correlation within various grades of birth asphyxia HIE, hypoxic-ischemic encephalopathy; vs, versus; Na+, sodium; K+, potassium; Ca+2, calcium

Parameters	Mild cases (%) (HIE I)	Moderate cases (%) (HIE II)	Severe cases (%) (HIE III)	Total cases (%)	HIE I vs HIE II P-value	HIE I vs HIE III P-value	HIE II vs HIE III P-value
Serum Na^+^							
Normal (130-150 mmol/l)	23(100)	51(59.3)	7(17.1)	81(54)	0.0005	<0.0001	<0.0001
Hypernatremia	0	7(8.1)	0	7 (4.7)	0.3495	_	0.1433
Hyponatremia	0	28(32.6)	34(82.9)	62(41.3)	0.0037	<0.0001	<0.0001
Serum K^+^							
Normal(3.5- 5.5 mmol/l)	12(52.2)	27(31.4)	1(2.4)	40(26.7)	0.0648	<0.0001	0.0006
Hyperkalemia	11 (47.8)	58(67.4)	39(95.2)	108(72)	0.080	<0.0001	0.0013
Hypokalemia	0	1(1.2)	1(2.4)	2(1.3)	0.6034	0.4503	0.5891
Serum Ca^2+^							
Normal	13(56.5)	19(22.1)	0	32(21.3)	< 0.0001	< 0.0001	0.0027
Hypocalcemia	10(43.5)	67(77.9)	41(100)	118(78.7)			
Serum urea							
Normal	20(86.9)	38(44.2)	4(9.8)	62(41.3)	0.0006	< 0.0001	0.0003
High	3(13.1)	48(55.8)	37(90.2)	88(58.7)			
Serum Creatinine							
Normal	23(100)	60(69.8)	4(9.8)	87(58)	0.006	< 0.0001	<0.0001
High	0	26(30.2)	37(90.2)	63(42)			

The degree of hyponatremia, hypocalcemia, and hyperkalemia (124.4±4.4 mmol/l, 0.83±0.09 mmol/l, 6.17± 0.89 mmol/l respectively) were significantly different between HIE III and HIE I (137.5±3.8 mmol/l, 1.05±0.19 mmol/l, 5±0.79 mmol/l, respectively). Similarly, serum urea and creatinine levels were significantly different between HIE I and HIE III (Table 3).

**Table 3:** Mean values of biochemical parameters in different stages of hypoxic-ischemic encephalopathy Statistical analysis, ANOVA, and post hoc test. HIE, hypoxic-ischemic encephalopathy; vs, versus; Na+, sodium; K+, potassium; Ca+2, calcium; mmol, millimole; mg, milligram; dl, decilitre; SD, standard deviation

Parameters	HIE-l	HIE-ll	HIE lll	HIE l vs HIE ll P-value	HIE l vs HIE lll P-value	HIE ll vs HIE ll P-value
Serum Na^+^ (mmol/l) ± SD	137.5±3.8	132.7±6.8	124.4±4.4	< 0.01	< 0.01	< 0.01
Serum K^+^ (mmol/l) ± SD	5.00±0.79	5.55±0.77	6.17±0.89	< 0.05	< 0.01	< 0.01
Serum Ca^+2^ (mmol/l) ± SD	1.06+ 0.17	0.86 + 0.15	0.83+ 0.09	<0.01	<0.001	>0.05
Serum urea (mg/dl) ± SD	26.75± 8.8	47.98± 24.7	89.38± 28.6	<0.01	<0.001	< 0.01
Serum creatinine (mg/dl) ± SD	0.70±0.16	0.94±0.54	2.41±0.89	>0.05 (NS)	< 0.01	< 0.01

Of 150 cases, 29 (19.3%) had died. Mortality among mild, moderate, and severe stages were zero, two (2.3%), and 27 (65.9%), respectively. It was found that mortality in HIE III was significant compared to HIE I and HIE II (p<0.01). Recovery with no abnormal movement/focal neurological deficit was seen in all HIE I (n=66, 76.7%), whereas neurological sequelae were observed in HIE II (n=18, 21%) and HIE III (n=14, 34.1%) cases. 

## Discussion

**Figure 1:**
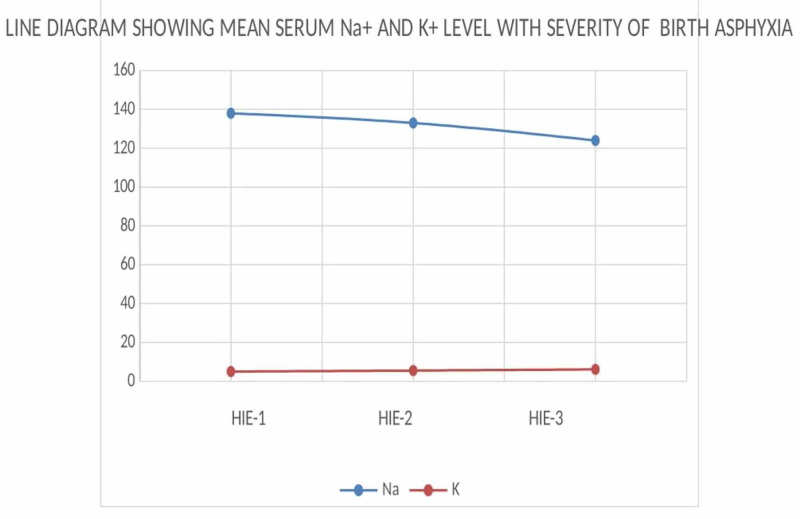
Line diagram showing the mean serum sodium (Na+) and potassium (K+) levels with the severity of birth asphyxia There is a progressive decrease in serum sodium (Na+) with increasing severity of birth asphyxia

Among the all asphyxiated babies, HIE II constituted the major portion (57.33%). So prompt identification of risk factors and biochemical derangements with timely intervention may prevent progression to severe grade and reasonably better neurological outcomes. The serum Na+, K+, and Ca+2 levels in the first 24 hours of life are between 134 and 146 mEq/L, 3.0 and 7.0 mEq/L, and 2 and 2.25 mmol/L, respectively [10]. Any change of sodium, potassium, and calcium levels in the blood beyond the normal range might cause seizures and metabolic abnormalities [14]. Calcium being the second messenger acts on various cofactors for many enzymatic activities and muscle contraction [14]. In this study, the degree of hyponatremia, hypocalcemia, and hyperkalemia was directly proportional to the degree of severity of HIE (Figure 1).

Hypoxia and ischemia cause neuronal injury and worsen cerebral edema due to maladaptation to the hyponatremic effect. So the quick and appropriate management of dyselectrolytemia reduces ischemic penumbra, seizure, and further CNS injury [14].

It was found that post-dated neonates are more likely to develop HIE II and HIE III (HIE II: 45.3%, HIE III: 53.7%) compared to HIE I (p<0.01). This is due to postdated pregnancy associated with meconium aspiration, respiratory distress syndrome, sepsis neonatorum, oligohydramnios, macrosomia, and fetal birth injury [15].

In this study, the largest number of birth asphyxia babies were from normal vaginal delivery but mostly with a mild (47.8%) to moderate (52.2%) degree. It may be assumed that vaginal delivery is a difficult process and may cause a prolonged second stage of labor. Other associated factors like breech presentation, macrosomia, and large size baby increase the severity of birth asphyxia. Previous studies in other parts of India and developing countries had similar findings [11,16]. Our study revealed that forceps/ventouse delivery may lead to severe birth asphyxia (p < 0.01), whereas LSCS is better and associated with HIE I (p < 0.001). Findings reported by Benedetto C et al. in Italy found that instrumental deliveries have the highest rate of short-term maternal and neonatal complications [17]. Usually, vacuum extraction is one of the safe practices to shorten the second stage of labor and the prevention of prolonged labor-related complications. A study from Japan proved successful vacuum‐assisted deliveries, as it shortened the duration of extraction and no complication for neonates [18]. That’s true for delivery in normal cases but babies delivered by forceps at a late stage, leading to HIE had unfavorable outcomes. Prolonged labor was the commonest risk factor in all three stages of asphyxia. The prolonged labor leads to dehydration and exhaustion, and the fetus becomes distressed [19]. It also contributes to maternal infection, neonatal infection, and intracranial hemorrhage ultimately develops birth asphyxia [7,20]. Hypothermia was associated with the majority (61%) of cases of HIE III. Very recently, therapeutic hypothermia is a very successful modality to improve the outcome for infants with hypoxic-ischemic encephalopathy by neuroprotective effects [21].

**Figure 2:**
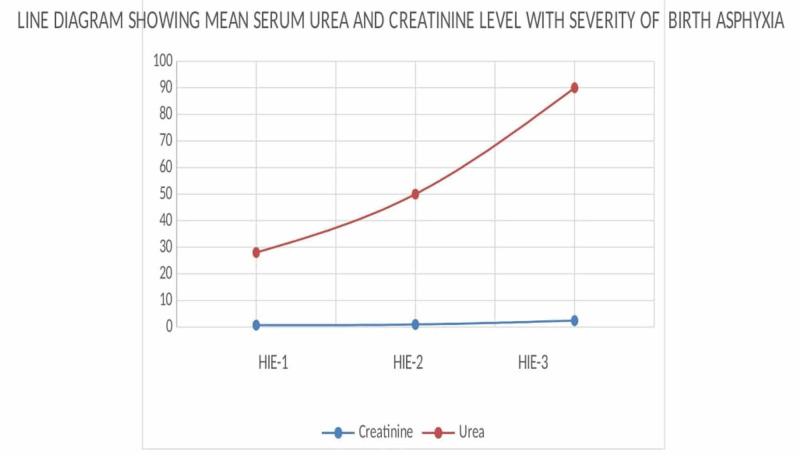
Line diagram showing the mean serum urea and creatinine levels with severity of birth asphyxia There is a progressive increase in serum urea and creatinine with increasing severity of birth asphyxia, but it is more marked in urea levels.

The kidney is the most common organ to be affected by HIE in the first 24 hours of life, and if hypoxia is not corrected later, irreversible cortical necrosis may occur [22]. So early diagnosis and appropriate fluid and electrolyte management is required for better outcomes for these newborns. Our study found serum urea and creatinine values proportionately increasing with stages of birth asphyxia (Figure 2).

These findings are comparable with other studies from India and Nepal [22-23]. Perinatal hypoxia causes acute tubular necrosis, renal vein thrombosis, and damage to nephrons. Thus, the sodium reabsorption capacity of the direct Coombs test (DCT) and more sodium loss in the urine causes hyponatremia. Further, birth asphyxia causes a syndrome of inappropriate antidiuretic hormone secretion (SIADH) and partial aldosterone resistance, leading to hyponatremia [16]. Hyponatremia leads to hypovolemia and further compromise of renal functions. The abnormal neurological finding is more prominent in HIE neonates (9/12;75%) having renal failure.

The average duration of hospital stay among mild, moderate, and severe stages of HIE babies were 3.9±0.7 days, 9.0±1.9 days, 17.6±3.5 days, respectively, indicating neonates with HIE III had longer hospitalization as compared to HIE I neonates (p < 0.05). Neonates with antiepileptic drug use or having focal neurological deficits were likely to have adverse outcomes and later may develop seizure disorder [24]. All the mildly asphyxiated neonates recovered uneventfully, whereas all the 29 (19.3%) cases who died belonged to the moderate and severe stages of birth asphyxia. There is a proportionate increase in mortality with the severity of HIE owing to the involvement of multiple systems [11]. The majority (59.3%) of HIE babies were discharged without focal neurological deficits. Similar reports were observed in other studies [11,25].

The present study has a few limitations. A large number of referral cases were coming from peripheral hospitals and the long duration of the journey may be responsible for the delay in receiving institutional treatment. These factors were responsible for prolonged labor, out-of-hospital deliveries, and more complications. Therapeutic hypothermia for infants ≥36 weeks gestational age (GA), with moderate-to-severe HIE, had not been used as a treatment modality for all, which might have an influence on the neurological outcome. Our findings may not be generalized to the community, however, due to the long duration of the study and a good number of cases, the findings of risk factors for HIE and correlation dyselectrolytemia can be used for planning the interventional strategy.

## Conclusions

Hypoxic-ischemic babies with dyselectrolytemia were found to have severe disease in our study group. The risk factors for birth asphyxia can be prevented by proper antenatal care and active and timely management during labor. Effective neonatal resuscitation and quick correction of electrolyte imbalances will help in the reduction of neonatal mortality and long-term neurological sequelae.

## References

[1] Lawn JE, Blencowe H, Waiswa P (2016). Stillbirths: rates, risk factors, and acceleration towards 2030. Lancet.

[2] NNPD Network (2005). NNPD Network, Indian Council of Medical Research, National Neonatology Forum. https://www.newbornwhocc.org/pdf/nnpd_report_2002-03.PDF.

[3] Leuthner SR, Das UG (2004). Low Apgar scores and the definition of birth asphyxia. Pediatr Clin North Am.

[4] Herrera CA, Silver RM (2016). Perinatal asphyxia from the obstetric standpoint: diagnosis and Interventions. Clin Perinatol.

[5] Scott H (1976). Outcome of very severe birth asphyxia. Arch Dis Child.

[6] Vashishtha VM (2009). The state of the world's children 2009: maternal health is the key to achieve MDGs 4 and 5. Indian Pediatr.

[7] Wosenu L, Worku AG, Teshome DF, Gelagay AA (2018). Determinants of birth asphyxia among live birth newborns in University of Gondar referral hospital, northwest Ethiopia: a case-control study. PLoS One.

[8] Chandra S, Ramji S, Thirupuram S (1997). Perinatal asphyxia: multivariate analysis of risk factors in hospital births. Indian Pediatr.

[9] Basu P, Som S, Das H, Choudhuri N (2010). Electrolyte status in birth asphyxia. Indian J Pediatr.

[10] Hansen AR, Soul JS (2012). Perinatal asphyxia and hypoxic-ischemic encephalopathy. Manual of Neonatal Care.

[11] Shah PS, Beyene J, To T, Ohlsson A, Perlman M (2006). Postasphyxial hypoxic-ischemic encephalopathy in neonates: outcome prediction rule within 4 hours of birth. Arch Pediatr Adolesc Med.

[12] Iii LCG, Leveno KJ, Burris J, Williams ML, Little BB (1989). Diagnosis of birth asphyxia on the basis of fetal pH, Apgar score, and newborn cerebral dysfunction. Am J Obstet Gynecol.

[13] Sarnat HB, Sarnat MS (1976). Neonatal encephalopathy following fetal distress. A clinical and electroencephalographic study. Arch Neurol.

[14] Castilla-Guerra L, del Carmen Fernández-Moreno M, López-Chozas JM, Fernández-Bolaños R (2006). Electrolytes disturbances and seizures. Epilepsia.

[15] Mehar V, Agarwal N, Agarwal A, Agarwal S, Dubey N, Kumawat H (2016). Meconium-stained amniotic fluid as a potential risk factor for perinatal asphyxia: a single-center experience. J Clin Neonatol.

[16] Thakur J, Bhatta NK, Singh RR, Poudel P, Lamsal M, Shakya A (2018). Prevalence of electrolyte disturbances in perinatal asphyxia: a prospective study. Ital J Pediatr.

[17] Benedetto C, Marozio L, Prandi G, Roccia A, Blefari S, Fabris C (2007). Short-term maternal and neonatal outcomes by mode of delivery. A case-controlled study. Eur J Obstet Gynecol Reprod Biol.

[18] Egami N, Muta R, Anami A, Koga H (2020). Impact of clinical practice guidelines for vacuum-assisted delivery on maternal and neonatal outcomes in Japan: a single-center observational study. J Obstet Gynaecol Res.

[19] Laughon SK, Berghella V, Reddy UM, Rajeshwari S, Zhaohui L, Hoffman MK (2014). Neonatal and maternal outcomes with a prolonged second stage of labor. Obstet Gynecol.

[20] Altman M, Sandström A, Petersson G, Frisell T, Cnattingius S, Stephansson O (2015). Prolonged second stage of labor is associated with low Apgar score. Eur J Epidemiol.

[21] Higgins RD (2005). Hypoxic ischemic encephalopathy and hypothermia: a critical look. Obstet Gynecol.

[22] Gupta BD, Sharma P, Bagla J, Parakh M, Soni JP (2005). Renal failure in asphyxiated neonates. Indian Pediatr.

[23] Shah G, Agrawal J, Mishra O, Chalise O (2013). Clinico-biochemical profile of neonates with birth asphyxia in Eastern Nepal. J Nepal Paediatr Soc.

[24] Das K, Das SK, Pradhan S, Sahoo PI, Mohakud NK, Swain A, Satpathy S (2020). Clinical feature and outcome of childhood status epilepticus in a teaching hospital, Odisha, India. Cureus.

[25] Sankar MJ, Natarajan CK, Das RR, Agarwal R, Chandrasekaran A, Paul VK (2016). When do newborns die? A systematic review of timing of overall and cause-specific neonatal deaths in developing countries. J Perinatol.

